# Intergenerational grounding of women’s environmental non-migration

**DOI:** 10.1007/s11111-025-00475-w

**Published:** 2025-01-22

**Authors:** Bishawjit Mallick, Julia van den Berg

**Affiliations:** Department of Human Geography and Spatial Planning, Faculty of Geoscience, Utrecht University, Utrecht, The Netherlands

**Keywords:** Intergenerational learning, Intellectual capital, Environmental non-migration, Women’s agency

## Abstract

This study examines the impact of intergenerational learning and intellectual capital on women’s voluntary decisions to remain in place despite environmental risks. By investigating how women experience the decision to stay through intergenerational knowledge transfer, we analyze the adaptability of communities facing climate-driven livelihood challenges and the intricate socio-ecological factors that tie individuals to their homes. Through life-story interviews with 70 women from 25 households in five environmental hazard-prone sites in Bangladesh, the study reveals nuanced patterns of traditional gender roles that both support and limit women’s autonomy in making mobility choices. Although mobility decisions vary across site and scale, systemic issues such as arranged child marriage, financial instability, (traditional) home-bound duties, male authority over mobility decisions, and gendered expectations consistently emerged as barriers to women’s (non-)migration, even when they aspired to leave. Thus, this research offers insights into gendered (non-)migration and its intergenerationality, which is inevitable in developing sustainable adaptation pathways.

**Supplementary Information:**

The online version contains supplementary material available at 10.1007/s11111-025-00475-w.

## Introduction

As environmental degradation and natural disasters intensify, migration often appears to be an inevitable option for those seeking refuge from deteriorating conditions (Black et al., 2011; Hunter et al., 2015; Szaboova et al., 2023). Yet, amid this narrative of displacement, many individuals exhibit a solid resolve to remain in their ancestral lands, resisting the conventional expectations of mobility in adversity (Farbotko et al., 2018; Mallick & Hunter, 2023). This is a deliberate decision taken voluntarily by many who are fighting against extreme events. These people are known as voluntary non-migrants (Mallick & Schanze, 2020), challenging simplistic views on climate-driven migration and highlighting the complex intersections of gender and environmental challenges (Furlong et al., 2022).

Women are the primary victims of climate change (Evertsen, 2024), bearing the brunt of climate-induced displacement, as evidenced by statistics showing they represent 80% of those displaced due to climate events (EU Gender Action Plan III, 2020). In disasters, women and children are up to 14 times more likely to die than men (UN Women, 2018). This heightened vulnerability stems from caregiving roles, as women often prioritize family well-being over personal safety, especially during crises (Bertana and Blanton, 2022; Perales et al., 2021). These outcomes are shaped by gender norms, deeply embedded in cultural, economic, and political contexts, and dictate distinct behavioral expectations for men and women, influencing access to resources, decision-making autonomy, and opportunities for education and employment. Their dependence on natural resources, home-bound labor, and limited education opportunities make women more vulnerable to climate change impacts and environmental hazards (Evertsen, 2024; Fry & Lei, 2021). These observations could reasonably imply that women would be the first to seek refuge elsewhere after environmental crises. However, empirical evidence suggests otherwise (Ayeb-Karlsson, 2020; Boas et al., 2022).

Women in many climate-affected regions exhibit remarkable resilience and resourcefulness, leveraging their intimate knowledge of local ecosystems and community networks to withstand environmental shocks and safeguard their families’ well-being (ibid). Their resilience is closely tied to a long-standing relationship with their environment, shaped by generational choices to remain despite the risks (Mallick & Hunter, 2024). Thus, a key element in understanding women’s resilience through non-migration is intricately connected to their historical relationship with their living environment.

Considerably, the choices of previous generations to remain in place have shaped the circumstances that influence current households’ livelihoods (ibid). In particular, women who face significant barriers, such as economic insecurity, early school withdrawal, and restricted decision-making power, must often rely on alternative strategies for resilience (Furlong et al., 2022). For instance, families exposed to environmental stressors over generations develop adaptive knowledge, passing it down intergenerationally (Mallick & Hunter, 2024). We refer to this process as “intergenerational learning”. Intergenerational learning embodies the transmission of knowledge, values, and traditions across different age groups within a family and community (Stephan, 2020). This dynamic process of intergenerational knowledge transmission serves as a cornerstone of cultural continuity, enabling the preservation of heritage and the cultivation of collective wisdom (Yembuu, 2021) and resilience-building throughout the generations in the face of evolving environmental and societal challenges. Understanding the role of intergenerational learning in shaping women’s non-migration decisions is vital for promoting gender equality, social mobility, and inclusive development in at-risk communities. It also takes on heightened significance as communities grapple with adapting to environmental shifts and mitigating their impact on future generations.

Well-researched factors such as place attachment (e.g., Blondin, 2021), social milieu (e.g., Upadhyay et al., 2023), or land ownership (e.g., Jennath & Paul, 2024) can influence women's choices to stay despite livelihood risks. However, there is still a lack of understanding about how intergenerational knowledge transfer and learning influences women’s decisions regarding non-migration (e.g., “gendered intergenerational learning” particularly in the context of environmental hazards) (Jennath & Paul, 2024; Mallick & Hunter, 2024). To address this gap, we critically examine gendered intergenerational learning and its role in shaping migration (non-migration) decision-making. Specifically, we explore how women’s non-migration decisions vary across generations and environmental hazards and how these variations are influenced by intergenerational learning.

Exploring these dynamics reveals how gender norms intersect with family dynamics, encouraging or inhibiting migration. Understanding these interlinked factors in navigating Twenty-first-century climate challenges is crucial for creating sustainable pathways that address women’s unique challenges and empower informed choices about migration.

The “State-of-the-art” section presents the relevant literature review, whereas the “Methodology” section describes the methodology and empirical tools. The “Results” section presents the results, while the “Discussion and outlook” section discusses the findings and concludes.

## State-of-the-art

### Interpreting environmental non-migration

**Fig. 1:**
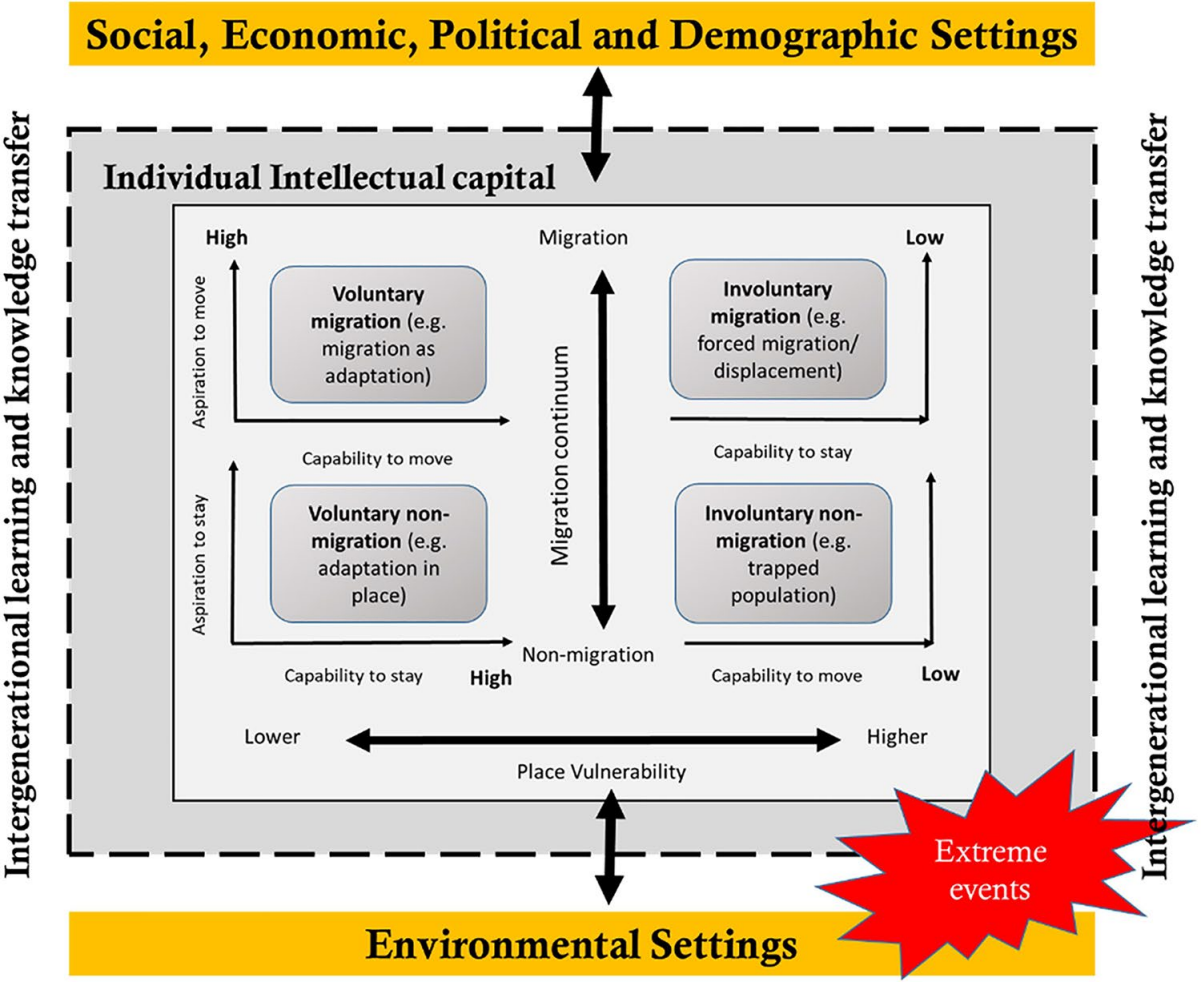
Analytical concept. Note. This figure presents the employed analytical framework. It demonstrates intellectual capital as key to explaining individual (im)mobility. Source: Authors’ illustration adopted from Mallick and Schanze’s framework (Mallick and Schanze, 2020), Hunter et al. (2015), and Mallick and Hunter (2024)

“Environmental non-migration” refers to the phenomenon where individuals or communities choose to stay in their current location despite facing environmental challenges such as natural disasters, climate change, pollution, or resource degradation (Mallick, 2023; Mallick and Hunter, 2023; Mallick & Schanze, 2020). This phenomenon is either voluntary or involuntary and is driven by mobility aspirations and capabilities (see Fig. 1 for the four individual-level categories of mobility). While voluntary non-migration involves a deliberate decision to remain in place despite adverse environmental conditions (high aspiration and capability to stay), involuntary non-migration occurs when people are unable or unwilling to leave due to socio-economic disadvantages or circumstances beyond their control (low capability to move and aspiration to stay) (Mallick, 2023). Figure 1 represents how people–place vulnerability can influence the voluntariness of people’s mobility. It often stems from a combination of factors such as attachment to place, economic considerations, and the perception of risk (Farbotko et al., 2018; Mallick, 2023; Mallick et al., 2023).

Voluntary and involuntary environmental non-migration represent complex responses to environmental challenges shaped by various factors, including cultural, economic, political, and social dynamics. By bringing this complexity into more detail by explaining its intergenerational and gendered dimensions, policymakers and practitioners can develop more effective strategies for supporting resilience, adaptation, and sustainable development in the face of environmental change.

### Intergenerational learnings and voluntary environmental non-migration

**Fig. 2:**
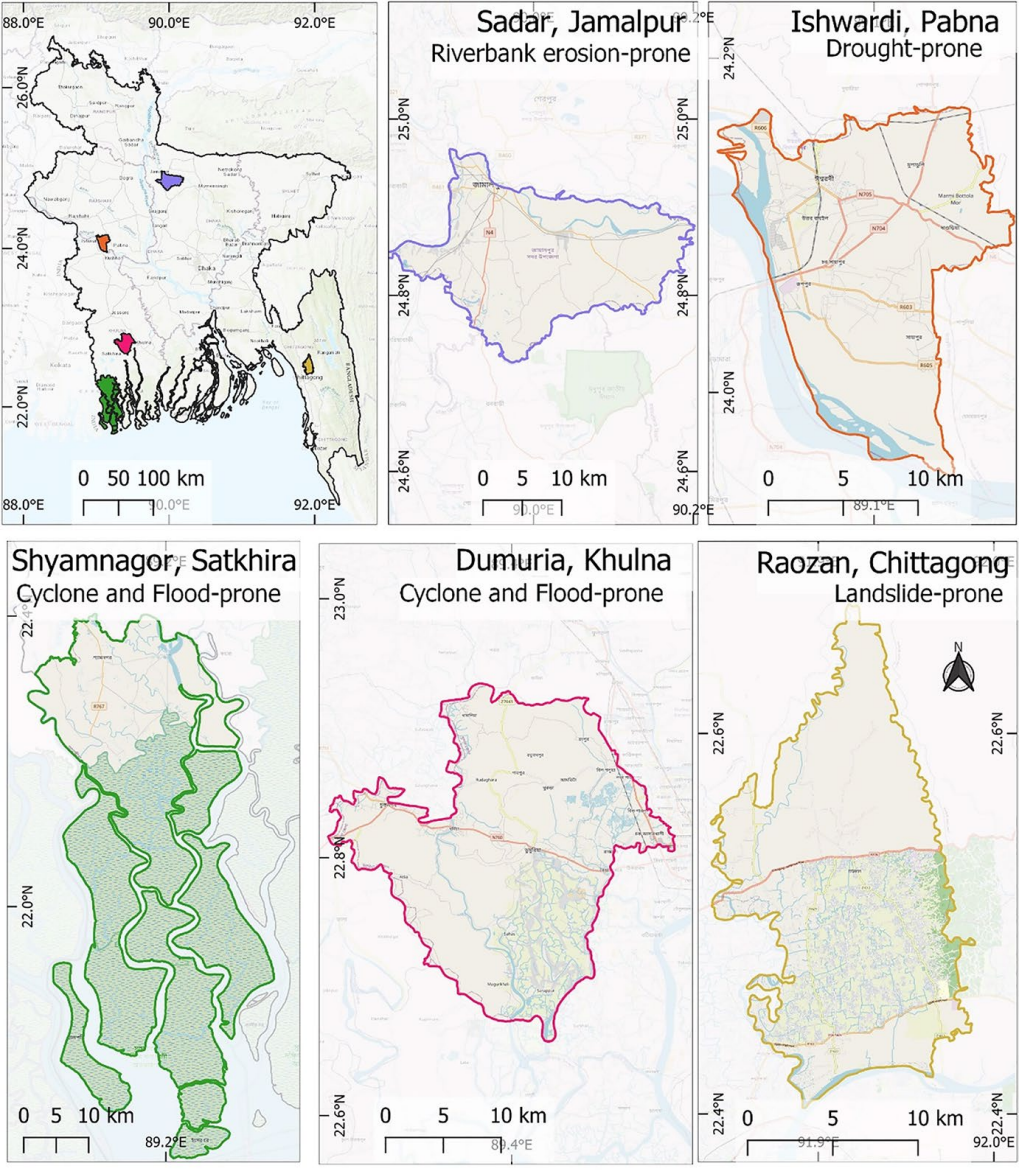
Study sites map. Note. This map presents the studied upazilas and their exposure to major hazards in Bangladesh. Source: Authors’ illustration

As previously stated, intergenerational learning refers to transferring knowledge, skills, and cultural norms from generation to generation (Spiteri, 2023). It is the most traditional method of pursuing lifelong learning and encompasses formal education and informal knowledge transmission within families, communities, and institutions (Mannion, 2016; Spiteri, 2023). It significantly affects individual mobility behaviors, which are often shaped by intellectual decision-making processes that are passed down through generations (Fig. 2). Mallick and Hunter (2024) examined how intergenerational learnings impact the migration aspirations and capabilities of households whose older generation experienced the 1970 Bhola cyclone, the deadliest tropical cyclone to date in the Bengal region. Their findings reveal that the intergenerational transmission of experiences and knowledge gained from this disaster enhances individuals’ capabilities and confidence to remain in place. Intergenerational learning is thus a crucial intellectual resource for making life decisions, including whether to migrate or stay. It plays a crucial role in shaping intellectual capital through preserving cultural heritage, passing down traditions, and facilitating socio-economic development (Spiteri, 2023; Yembuu, 2021). Intellectual capital is attributed to sociocultural, spatial, and individual factors and encompasses the knowledge, expertise, and innovative capabilities embedded within individuals, organizations, and societies. It includes human capital (individual skills and capabilities), structural capital (organizational processes and systems), and relational capital (networks and relationships). Intellectual capital—the collective reservoir of knowledge, skills, and innovation embedded within individuals—is at the heart of resilience. It is intersectional, spatial, and transferred intergenerationally. By nurturing such intellectual capital of different generations, communities may equip themselves with the tools to anticipate, respond to, and thrive amidst climate-related challenges (Mallick & Hunter, 2024). In addition, intergenerational knowledge transfers and intellectual capital hinge on the intersection of people’s identities, positions, and dominant social structures (Levac et al., 2018). However, the gendered dimensions of this intergenerational intellectual capital may produce disparities and opportunities within familial and societal roles, and can be important sources of diversities in intergenerational knowledge transfer (Andrijevic et al., 2020; Furlong et al., 2022; Mallick & Hunter, 2024).

### Gender, intergenerational knowledge, and mobility

Gender attitudes, norms, and roles are internalized and transmitted across generations, reinforcing traditional gender roles and ideologies (Akhter et al., 2023; Perales et al., 2021). Gender ideology and other identity scripts are shaped by and performed within an individual’s socio-environmental context (Lama et al., 2020). In Bangladesh, power dynamics and patriarchy dictate distinct gender roles for men and women in both public and private spheres (Furlong et al., 2022). Despite efforts to delay the age of marriage, girls in Bangladesh continue to marry at relatively young ages. Research shows that women’s participation in education declines after marriage, as many drop out of school during this transition (Chan et al., 2022; Lina, 2023). However, women’s education is crucial for enhancing broader human development outcomes, including increased earning potential for women, improved family decision-making ability, and better overall family health (ibid.). Moreover, equipping women with the necessary skills and competencies through education enhances their ability to lead, participate, and make informed decisions. Many of these skills and competencies are pertinent in addressing the challenges of climate change, thereby improving climate adaptation and resilience within families and communities (Fry & Lei, 2021). However, in Bangladesh, a country with a dominant patriarchal system, many household structures are characterized by a male dominance of control, power, and decision-making authority while restricting women’s agency, socio-economic roles, activities, and education opportunities (Akhter et al., 2023). As Furlong et al. (2022) state, “Gender shapes immobility and vice versa” (p. 13). Mobility decisions (i.e., migration or non-migration as climate adaptation) are thus reinforced through sociocultural traditions and expectations, which are transferred intergenerationally (Furlong et al., 2022; Mallick & Hunter, 2024). Further, male decision-making authority and experiences of particular climate disasters by prior generations may shape the mobility outcomes of current generations. This occurs through transferring hazard-specific adaptive knowledge and the enforcement of mobility decision consequences shaped by intergenerational knowledge, gender norms, and societal scripts. Understanding these intersections is an important knowledge gap we addressed in this research.

### Analytical concept

We propose an analytical concept (see Fig. 1 and Table 1 for details of the components) that defines the societal and individual-level factors contributing to individual-level mobility outcomes in an intergenerational context. The analytical concept presents the mobility continuum, from migration to non-migration decisions, influenced by learning and knowledge transfers through the individual attributable context (locality, intersectionality, and intergenerational), environmental settings, and social, economic, political, and demographic settings, ultimately shaping individual intellectual capital. These attributes, settings, and capital affect the individual’s capability and aspiration to move or stay. Non-migration is a mobility outcome shaped by these conditions. Its processes and consequences are contingent on societal and individual-level factors. Besides, the environmental stress and extreme events of a place ultimately contribute to one’s aspiration and, to a further extent, capability to move or stay. Environmental stress, encompassing both slow- and rapid-onset events, may aggravate livelihood challenges, leading some to decide hat migrating is necessary. Environmental stressors may also impair one’s capability to migrate. Here, climatic risk or extreme events can both increase incentives for people to migrate but can also aggravate their livelihood vulnerability if they depend on natural resources (Blondin, 2021; Nabong et al., 2023).

**Table 1:** Components of the analytical concept

Components	Factors that define the components	Definitions of the factors	Example from literature
Individual intellectual capital—intergenerational knowledge and learning transfer	Intergenerational	The transfer of intellectual capital from one generation to another	Ayalon, L., Roy, S., Aloni, O., and Keating, N. (2022). A scoping review of research on older people and intergenerational relations in the context of climate change. *The Gerontologist, 63*(5), 945–958
	Intersectional	The role and intersection of people’s identities, positions, and dominant social structures in shaping intellectual capital	Perales et al. (2021). Mothers, fathers and the intergenerational transmission of gender ideology. *Social Science Research, 99,* 102597
	Spatial	The spatial context in which individuals are positioned shapes the contents of intellectual capital. This refers to societal as well as environmental context	Cristancho, S., and Vining, J. (2009). Perceived intergenerational differences in the transmission of traditional ecological knowledge (TEK) in two indigenous groups from Colombia and Guatemala. *Culture & Psychology*, *15*(2), 229–254
Migration continuum—migration/(Non-)migration decisions	Capability to move	The synthesis of personal, societal, and environmental factors and tools that allow individuals to migrate to another place to find residency	Evertsen (2024). Gender, environment and migration in Bangladesh. *Climate And Development, 12*(1), 12–22
	Capability to stay	The synthesis of personal, societal, and environmental factors and tools that allow individuals to remain in their current place of residency	Biswas, B., and Mallick, B. (2020). Livelihood diversification as key to long-term non-migration: evidence from coastal Bangladesh. *Environment, Development And Sustainability, 23*(6), 8924–8948
	Aspiration to move	The individual’s desire to migrate to another place to find residency	Mallick and Schanze (2020). Trapped or voluntary? Non-migration despite climate risks. *Sustainability, 12*(11), 4718
	Aspiration to stay	The individual’s desire to remain in their current place of residency	Furlong et al. (2022). Gendered (im)mobility: emotional decisions of staying in the context of climate risks in Bangladesh*. Regional Environmental Change, 22*(4)
Collective engagement—drivers	Social	Social conditions influencing (non-)migration decisions	Castelli, F. (2018). Drivers of migration: why do people move? *Journal Of Travel Medicine, 25*(1). 10.1093/jtm/tay040
	Economic	Economic conditions influencing (non-)migration decisions	
	Political	Political conditions influencing (non-)migration decisions	
	Demographic	Demographic conditions influencing (non-)migration decisions	
Environmental settings—place vulnerability^1^	Cyclone		Poncelet, A et al. (2010). A country made for disasters: environmental vulnerability and forced migration in Bangladesh. In *Springer eBooks* (pp. 211–222)
	Drought		Kabir, M. E et al. (2017). Seasonal drought thresholds and internal migration for adaptation: lessons from Northern Bangladesh. In *Springer eBooks* (pp. 167–189)
	Flood		Chumky, T et al. (2022). Disaster-induced migration types and patterns, drivers, and impact: a union-level study in Bangladesh. *World Development Sustainability, 1*, 100,013
	Landslide		Sarker, A. A., and Rashid, A. K. M. M. (2013). Landslide and flashflood in Bangladesh. In *Disaster risk reduction* (pp. 165–189)
	River erosion		Mollah, T. H., and Ferdaush, J. (2015). Riverbank erosion, population migration and rural vulnerability in Bangladesh (a case study on Kazipur Upazila at Sirajgonj District). *Environment And Ecology Research*, 3(5), 125–131

This table presents definitions and research examples on the components as presented in the analytical concept (see Fig. 1)

^1^Definitions for each environmental hazard are not provided, as we deem them unnecessary due to their widespread recognition and understanding within the research context

Figure 1 highlights that societal and environmental contexts together shape intellectual knowledge at the individual level, which can vary based on intersectional factors such as gender and geography, as well as across intergenerational and gendered outcomes. Intergenerational learning and knowledge transfers also determine individual-level mobility outcomes (Mallick & Hunter, 2024). Because intellectual capital constitutes formal (institutional) and informal (familial/relational) learning structures, it is also to be gendered through gender-specific intergenerational knowledge transfers as befitting dominant societal norms, roles, and ideologies. These intergenerationally transferred ideas shape and restrict women’s physical and social positions, often being home-bound to an unpaid, caretaking position and having dropped out of secondary-level education (Akhter et al., 2023). Reinforcing sociocultural traditions and expectations shapes mobility decisions (Furlong et al., 2022). Within the household, male dominance of decision-making authority can restrict the woman’s ability to migrate, even if she has high aspirations. However, generational expectations and traditions can also shape women’s aspirations of staying at home. Passed-down traditional gender norms and ideologies can thus contribute to unequal mobility capabilities and aspirations.

The synthesis of these factors culminates in four individual-level categories of mobility: (i) voluntary migration—high capability and aspiration to move; (ii) involuntary migration—low capability to stay and a low aspiration to move but forced to move; (iii) voluntary non-migration—high capability and aspiration to stay; and (iv) involuntary non-migration—low capability to move and a low aspiration to stay, but forced to stay. In this study, we examine these four categories of mobility as affected by women’s intergenerational learning and intellectual capital and spatial stratification across environmental hazards (see Table 1 for the factors and components of the concept).

## Methodology

### Study sites

We employ an inductive research approach through the life-story method (Goodson & Sikes, 2016). Spatially diversifying our analysis of the intellectual capital and mobility decision-making of the study’s participants vis-à-vis different environmental hazards, we selected five specific environmental hazard-prone *upazilas* (administrative regions) in Bangladesh. The study sites are presented in Fig. 2. The selected upazilas are each known for their vulnerability to certain hazards. For example, Khulna and Satkhira are coastal areas frequently affected by cyclones due to their proximity to the Bay of Bengal (BDP 2100; Dasgupta et al. 2009). Natore and Jamalpur are flood-prone areas, while Chattogram is known for landslides due to its hilly terrain (ibid). Studying these areas provides insights into the most affected regions and the severity of impacts. The upazilas are spread across different regions of Bangladesh, including the southwest (Khulna and Satkhira), northwest (Natore), central (Jamalpur), and southeast (Chattogram). The geographical diversity of the study helps in understanding how different regions with varying topographies and climates experience and respond to environmental hazards. The findings from these diverse and hazard-prone areas can help inform Bangladesh’s regional and national disaster management policies. Lessons learned from these areas can be applied to other regions facing similar hazards, making the findings more broadly applicable across Bangladesh and possibly in similar contexts globally.

### Data and analysis

We collected the data from April to May 2023 with an inductive qualitative life-story approach, focusing on the intergenerational livelihoods of women. We recruited four field research assistants locally, who validated the interview guides and conducted semi-structured, in-depth interviews in Bengali, the local language. These life-story interviews were designed to collect intergenerational livelihood resilience perspectives on (a) the climate change–induced impacts on their livelihood conditions over the generations their family has been living there, (b) the impacts of these changes on the livelihood of the participants and their household members, (c) their strategies to overcome these impacts, and (d) their experience, knowledge, and views on climate change.

We selected five households from each hazard-specific village. Ultimately, three generations of women (i.e., grandmother, mother, daughter) from 25 households were interviewed in each of the five selected study villages. We omitted five interviews that we deemed linguistically incomprehensible or strayed too far from the guide. Thus, we have included seventy participants in the study. The participants were selected through snowball sampling. We interviewed women from all three generations, but with a minimum age of 16 years, according to the approved IRB. Supplementary Table S1 provides a list of participant information.

After the interviews, the field research assistants manually transcribed the interviews. The transcriptions were then translated into English for the researchers to analyze. The raw data present in the transcripts, in turn, was coded manually and in NVivo employing a combination of inductive and deductive approaches. In the inductive approach, themes were derived from the data itself without predefined categories, while the deductive approach involved applying existing theories to categorize the data. This dual coding strategy allowed for a comprehensive analysis that integrated both emergent and theory-driven insights. The coding process culminated in establishing a code book, which can be found in Supplementary Table S2. When the code book was established and coding completed, the data were analyzed and differentiated the data based on the three key outcomes: (i) intergenerational, which focused on the transmission of knowledge and practices across generations, (ii) intersectional, which examined how overlapping identities such as gender, age, and socio-economic status shaped the experiences and decisions, and (iii) spatial, which considered how location and mobility influenced adaptation strategies. By breaking down the analysis into these specific dimensions, the study provided a more comprehensive understanding of the complex factors at play. The following section will discuss the results of this data analysis.

## Results

### Involuntary vs voluntary (non-)migration

Mobility decisions differ across households, generations, and sites. Outcomes depend on (inter)personal capabilities, aspirations, and societal and ecological drivers, as described in the analytical concept (Fig. 1).

#### Involuntary non-migration

Although for many interviewed women, free movement is limited to the familiar spaces of their home(town) or is temporary in nature (e.g., for visiting family purposes), we recorded numerous accounts of high aspirations to move. The underlying motivations for women’s migration aspirations differ per household generation. Many third-generation participants wished to move to bigger cities or abroad for study opportunities. Some second-generation participants had aspirations to move for employment opportunities, to climatically safer regions (mostly from Jamalpur), to buy more fertile land, or for their children’s future. Notably, participants who live in areas prone to flooding and river erosion more often expressed an aspiration to move than participants in other study sites. Another participant, Shovna, who lives in Natore, details that she has a deep aspiration to move for the future of her (grand)children:

I spend my days in distress, hoping that if they can somehow manage to build a little future, maybe they will be able to find a job. If they pass college exams with good results, they might get a job in a private or government company.

Shovna is a minority of first generations who expressed a desire to move. Despite such aspirations to move, most interviewed women do not have the financial capital to do so. Rupamadhuriya, a second-generation participant, states, “If we had enough money, we would go to another place and leave. I don’t like this place”. Moreover, female migration experiences, capabilities, and aspirations depend on male decision-making authority. Families’ livelihoods hinge on the men in the household for financial security. Most men in interviewed households are the primary breadwinners; for some households, they are the sole breadwinners. Moving to another place without securing employment beforehand would thus (temporarily) cause a loss of livelihood. Puthi, a second-generation woman, indicates, “He doesn’t want to go. What will he do if we get out of here? We have income here, and everything is here; that’s why he doesn’t want to go.” However, not all families are immobile and passive in their mobility decisions. Some men engage in seasonal labor migration or have migrated with their families for employment opportunities, often to Dhaka, neighboring cities, or abroad. Although many women aspired to move, others explicitly stated they desired to remain.

#### Voluntary non-migration

Many first- and second-generation participants articulated the prospect of dying in their current house due to their weak physical health or attachment to their home. As Nitya, a first-generation participant from Khulna, states, “Where will I go? The destination is the grave.” Besides this wish, many women expressed a deep place attachment that overshadowed, in some cases, their (climate) vulnerable livelihood at their current place of residence. Never having left their (in-laws’) house, being familiar with the environment and the people, and knowing no other people in other places prevent aspirations to move. Some women named their place of residency the “homeland” or “my country”, evidently detailing a sense of belonging or loyalty. As Nitya says, “There is no country like my own country.” This sense of belonging and deep place attachment cognitively manifests in the aspiration to stay.

Similarly, Kavya, who lives in Natore, expresses that “we are people of the river”, articulating a deep sense of belonging to the Padma River. Other reported reasons for voluntary non-migration were caregiving tasks, (stable) employment, fear of property loss, and social ties. Most households’ voluntary non-migration reflected a combination of factors rather than a single cause.

Concurrently, women’s strong connection to home is often rooted in accepting home as belonging to their husbands. Their husband’s desire to remain is frequently internalized in the low aspiration to move. Esha, a first-generation participant from Satkhira, exemplifies this notion, stating, “We don’t have the ability, so there is no thinking either.” Although Esha initially dreams about migrating, she later expresses that she wants to stay. Similarly, when asked about leaving when having the opportunity, Aarti,^1^ a first-generation woman from Satkhira, states, “There is no point in thinking about it. The men of the house did not want to go. As a woman, I can’t go alone even if I want to go.” It seems worth explicitly recognizing that few “voluntary” non-migrants seem like they actually want to stay according to these quotes.

Non-migration aspirations and capabilities (categories: low–moderate–high), stratified across generation and hazard, are presented in Table 2. We want to emphasize that these findings are based on qualitative life-story interviews and may not have statistical significance. It claims the need for further explorations using more extensive datasets. Categorization is based on the prevalence of expressed aspirations and capabilities to move per participant (generation) vis- à -vis study site (hazard).

**Table 2:** Migration aspirations and capabilities

Hazard^1^	Generation					
	First		Second		Third	
	Aspiration	Capability	Aspiration	Capability	Aspiration	Capability
Cyclone	Low	Low	Moderate	Moderate	Moderate	Moderate
Drought	Low	Low	Low	Low	Moderate	Moderate
Flood	Low	Low	Moderate	Moderate	High	Moderate
Landslide	Low	Low	Low	Low	Moderate	Moderate
River erosion	Low	Low	Moderate	Moderate	High	Moderate

This table demonstrates participants’ aspirations and capabilities to move and stratify across environmental hazards and generation, as experienced in the study sites. See Table 3 for examples of each aspiration and capability category

^1^The occurrence of each hazard according to the study site can be found in the “Study sites” section

^2^Definitions of the scale categories are as follows: Low = this category includes individuals who expressed a low aspiration and/or capability to move (high non-migration aspirations and/or capabilities). Moderate = this category includes individuals expressing indifferent, doubtful, or uncertain aspirations and/or moving capabilities. High = this category includes individuals who expressed a high aspiration and/or capability to move (low non-migration aspirations and/or capabilities)

#### Environmental hazards and mobility outcomes

In some cases, whereas women expressed the vulnerability of their house to certain climate risks such as river erosion, their husbands often felt a deep attachment to and rootedness in their childhood home and refused to move to a safer region. Families were forced to move only when houses were too damaged or washed away. However, these movements for most interviewed households were temporary and limited within the boundaries of the *upazila*.

### Intergenerational and intersectional staying outcome by hazards

(In)voluntary non-migration is strongly affected by the place of residency and environmental hazards. Therefore, we selected the communities based on their propensity to specific hazards.

#### Landslides

Chottogram suffers from landslides, a mass of rocks, earth, or debris that slides down a slope after heavy rainfalls. Kamini, a third-generation participant, explains, “My grandmother said that if it rains continuously for seven days, then the ground becomes soft. Then, there is a high possibility of falling, and it even happens. […] Soil fell down.” Although most households have experienced landslides, none of the interviewed households were displaced because of these events. Some women indicated their houses were not at risk, and others said they should protect their homes. Kamini explains that the most considerable risk of the landslides for her family is not the landslide itself but the trees that fall as a result, “If another storm or rainfall comes, the trees will fall. Then, at that moment, everyone will cut it down as a precaution.” By taking precautions such as cutting trees and digging soil to make way for surplus water after heavy rainfall, landslides have never been fatal or a cause of forced displacement for the interviewed households. Therefore, none of the interviewed women in Chottogram expressed they desired to migrate due to environmental factors.

#### Cyclones

Most women, across all generations, in Khulna and Satkhira, have had numerous direct experiences with cyclones. They reported that homes were often damaged, swept away, or inundated during such events. Documented consequences of these inundations include food scarcity, salinization of drinking water, waterborne diseases, and forced displacement. Diti, a first-generation participant living in Satkhira, describes how, during Cyclone Aila in 2009, her “house was destroyed. We could not stay. It came so suddenly, we couldn’t take anything.” This experience was echoed by other women who had no place to live after the destruction of their house and had to live on the street. “Everyone lived on the road, there was no other accommodation. I was there for about 2–3 months”, as told by Mita, a second-generation participant who lives in Satkhira. Other households sought temporary refuge in cyclone shelters. Shelters provided a place to stay during and in the aftermath of cyclones. However, they were often poorly maintained, lacked sanitation, and were experienced by some as unsafe for women. In many cases, after the water from the storm surge had subsided and houses had been repaired, most women returned to their in-laws’ homes despite the exposure and vulnerability to climate risks. Living in their in-laws’ household, they often have little agency in mobility decision-making, as their in-laws are deeply attached to their home and their husbands are similarly rooted in their childhood residence.

#### Floods and river erosion

In Jamalpur and Natore, many households suffer from annual floods and river erosion, leading to the recurrent loss of property, livelihood, and security. Under these circumstances, many households were forcefully displaced, with frequencies of displacement ranging from one to an estimated fifty times over a lifetime. Indeed, many women detail how river erosion and flooding have been a part of their lives since childhood. The 71-year-old Shovna, who grew up 20 km from Natore, remembers from her childhood, “We moved to a dry place or to my granny’s house, which was not affected. When the water was gone, we would be back at home.” Families with sufficient financial capital moved away from erosion- and flood-prone areas, while those who stayed behind were still exposed and vulnerable to these hazards. Chayna, a 40-year-old homemaker living in Jamalpur, explains the socio-economic divisions consequential to continuous river erosion: “Those who could buy land for themselves. Those who can’t are staying by the roads. These are government lands.” Families on these government lands build their own “huts”; provisional houses often made from mud and that are exceptionally vulnerable to floods, erosion, and storms.

For many interviewed households, forced migration is a part of life. However, similar to Khulna and Satkhira, migration for interviewed households was primarily confined within the boundaries of the city. Additionally, when the damage to their in-laws’ house was reparable, households returned home for reasons similar to those reported by women in Khulna and Satkhira, where migration was often temporary. In these regions, families tend to return to their original homes once conditions improve, driven by the need to restore normalcy, maintain community ties, and manage livelihoods, reflecting a pattern of short-term migration rather than permanent relocation.

#### Drought

In addition to floods, participants in Natore suffer from annual droughts that cause food and water shortages and a lack of employment opportunities. Due to its temporary nature and adaptive measures, such as installing deep wells or temporarily working fields in other places, none of the interviewed households reported a need to migrate due to these droughts.

#### Mobility outcomes and intergenerational learnings

Cyclones, floods, and river erosions cause forced displacements. When it is safe, however, most households choose to return to their hometown, even though they recognize the exposure and vulnerability to climate risks. As women often hold a passive position in mobility decision-making, returning home can be a consequence of male place attachment, fear of property loss, employment, or education of the children. Women generally live in their in-laws’ homes, further limiting their decision-making authority. Women’s capability, aspiration, or obligation to remain in place transcends all climate hazards. Residing in areas vulnerable to such hazards necessitates developing and implementing climate-adaptive strategies to protect livelihoods. Families that have endured generations of exposure to floods, cyclones, landslides, river erosions, and droughts have cultivated strategies to stay put in these environments, with this knowledge being passed down intergenerationally. This transfer of adaptation strategies is closely linked to the gendered division of labor within households. Women develop and refine adaptive caretaking practices, which they then pass on to their daughters. Despite ongoing risks, these strategies enable families to sustain their livelihoods in hazard-prone areas. The non-migration decisions of households reinforce intergenerational transfers of knowledge and make it possible for families to remain in these regions, relying on these resilience methods.

### Gendered intergenerational learning

Knowledge transfers and intellectual capital are partly a result of intergenerational and intersectional stratified learning categories as shaped by societal norms and expectations. While general socialization focuses on reinforcing normative gender roles and expectations within a societal context, intergenerational learning highlights the active exchange of context-specific experiences and practices, which may either reinforce or challenge traditional roles based on adaptive needs. This process often involves a dynamic interplay between evolving gender norms and the practical requirements of resilience and adaptation. Further, gendered intergenerational learning differs from general socialization into gender roles by emphasizing the transfer of specific knowledge, skills, and adaptive strategies between generations, particularly in response to environmental or societal challenges. This subsection examines intellectual capital on these intertwined factors in the context of female livelihood resilience.

#### Shifts in generational roles and aspirations

Many women were raised with familial expectations and duties preparing them for a domestic role encompassing unpaid, home-bound labor such as cooking, cleaning, and caregiving. Most first- and second-generation participants, as well as some third-generation participants, were married off between the ages of 10–16 as child marriage was, and for some still is, a customary practice. It is expected for women to move to their in-laws’ houses after marriage, either until death or until their husband decides to move, thereby taking away their agency and freedom of mobility. This means that most interviewed women, across all study sites, were forced to leave their home(town) at an early age. As a result, most had to quit their education as they had to meet certain expectations of their (gendered) role in the household. In addition, women are expected to bear children after marriage, which was, for many participants, another reason for dropping out of school. Especially for first- and second-generation women, it was not common for girls to continue or pursue any education due to the poor accessibility of schools, their duties with helping their parents around the house, low financial capital, or their role in the house after marriage. The discontinuation of formal education emphasizes the importance of learning the tasks that align with becoming a “good wife” and caregiver. Nowadays, expectations are changing as most third-generation participants are enrolled in school and aspire to pursue a higher degree for specific employment opportunities. Thirty-five-year-old Joti expresses her aspirations for her daughter’s future:

It’s different for girls. Girls get very little education. They live at the in-laws’ house. I couldn’t continue my studies because I got married at an early age, so I don’t want the same for my daughter.

Being enrolled in school, girls are less involved in homemaking tasks by their elders than when their (grand)mothers were their age. However, the perspective of marrying their daughters persists in many households, and the expectation of relocating to the in-laws’ house and abiding by their husband’s wishes is reinforced in these households for girls already from a young age. The 11-year-old Roudri expresses, “I will build a house when I grow up. Then, if the sir [husband] asks me to go anywhere, I will go”. Even though these patterns are slowly changing, women and girls like Roudri are still expected to learn from their parents and elders to remain in their in-laws’ houses after marriage.

#### Intergenerational learning and intellectual capital

Although traditional gender roles are typically linked to unpaid, domestic responsibilities, the direct and indirect financial contributions of women in many interviewed households play a crucial role in the household. Many women are responsible for growing homegrown produce, processing animal products, and making goods their husbands can sell. Other women work as *biri* (hand-rolled cigarette makers), sew fishing nets and clothes, work in jute mills, and on other people’s land and homes. Moreover, intersecting with traditional gender power structures and decision-making authority, women’s immobility affects their vulnerability to climate risks. Women must have resources to protect their household’s livelihood during climate hazards. By cultivating and preparing homegrown crops and livestock products, households are better equipped with a stockpile of dried and preserved food, ensuring resilience during climate-related disasters. Women in Jamalpur explained that they preserve food such as rice and wheat flour in preparation for the annual floods. Additionally, loose items and furniture are raised from the floor to stay dry. Such strategies are mostly passed tacitly and through observation. Chayna, a 40-year-old homemaker who lives in Jamalpur, describes how she learned from her mother to make preparations for floods:

I learned because my mother used to do it. Before the flood approached, my mother used to store rice and lentils. Then, she arranged firewood and stoves. We’ve learned these. We also do the same because if we don’t take measures, what will my children eat during the flood?

Similarly, Soma, a 70-year-old woman from Jamalpur, describes her observations of her mother’s cooking strategies during a flood, “There was a wooden chair back then. She put bricks under the chair to lift it up. Then, she put the stove on the chair and cooked”. Soma now employs the same strategy of cooking on an elevated chair to prepare meals for her family during floods. She has passed this method on to her daughter-in-law, who utilizes the elevated cooking chair. Women in other study sites who face various natural hazards, such as river erosion and tropical cyclones, provided similar accounts of knowledge transfer across generations regarding preparedness strategies. A 13-year-old third-generation participant from Jamalpur explains that she learned from her mother to fetch water from the tube well and store dry food in preparation for a flood. In Khulna, the practice of seeking safety in cyclone shelters during tropical storms is often informed by intergenerational knowledge and experiences. Older family members, who have lived through previous storms, play a crucial role in guiding younger generations on when and how to evacuate, as well as how to prepare for the storm. This intergenerational exchange of adaptive strategies not only ensures physical safety but also reinforces community resilience by embedding life-saving practices into the cultural fabric of families over time. From a young age, children learn that these places provide them with protection and basic needs.

#### Intergenerational mobility and learning

Two gendered intergenerational mobility and learning trends emerge. Some women want to break the intergenerational cycle of forced migration through marriage, followed by forced non-migration by domestic responsibilities by sending their daughters away for educational purposes. On the other hand, other women want to marry their daughters off, thereby continuing this immobility cycle through the next generation. Regardless of women’s aspirations for their daughters, those who live in areas susceptible to climate hazards pass down adaptive knowledge orally, through repetition, and via imitation. See Table 3 for participant quotes touching on intellectual capital and migration outcomes, stratified by generation.

**Table 3:** Illustrations of the analytical concept factors

Component		Generation		
		First	Second	Third
Intergenerational intellectual capital		“She [mother] put bricks under the chair to lift it. Then, she put the stove on the chair and cooked” (J-F7-G1-160623-F)^1^	“I watched that my mother used to place chicken and ducks on an elevated place which she reared [during a flood]” (J-F7-G2-160623-F)	“They [family members] fold items in the house and keep it on the bed. They also store dry food” (J-F7-G3-160623-F)
Intersectional intellectual capital		“That time girls didn’t use to study much” (I-F2-G1-120523-F)	“Girls get very little education. They live at their in-laws’ house” (I-F1-G2-110523-F)	“After my marriage, my husband got a job in Dhaka. That’s why I can’t continue my education anymore” (I-F5-G3-010523-M)
Spatial intellectual capital		“At that time, I didn’t go to school because it was far away” (J-F2-G1-0523-M)	“Our brothers didn’t allow women outside of the home. They cultivated the crops” (I-F5-G2-010523-M)	“School is closed at that time. Water rises in the school. It causes studying difficulties” (J-F3-G3-0523-M)
Capability to stay	Low	“We suffer a lot during a flood. So, if we had the ability we would’ve moved” (J-F9-G1-15023-F)	“I feel like going, but I can’t. I will need more money to build the house” (J-F5-G2-160623-S)	“When my brother earns enough money, we will sell this land and move to an elevated place” (J-F8-G3-170623-F)
	Moderate	“They’ve [her son and his family provided this place to stay. If they go, I will also leave” (J-F9-G1-150623-F)	“If this land had eroded, then I don’t have the brain to stay anymore. I don’t have land, then I have to go” (I-F3-G2-110523-S)	“If my father and mother go, I will go” (K-F3-G3-060523-T)
	High	“My area is good. It is good by the grace of God” (I-F2-G1-120523-F)	“I don’t feel the need to move elsewhere for these reasons. If there is a problem, we wait for a few days for it to be solved” (I-F1-G2-110523-F)	“We can have all kinds of facilities if we want to. Then it will be peaceful to live here rather than living in the city” (C-F3-G3-210523-F)
Aspiration to stay	Low	“If I have money, then I will move to another place” “J-F9-G1-150623-F)	“I wish to live in better conditions. I don’t want to live in such heat, water problems, water logging” (I-F1-G2-110523-F	“This is a village area. When I go to study, I will leave here” (J-F6-G3-170623-S)
	Moderate	“If Allah allows, I will reside here. Otherwise, if they [her son and his family] go, I will also leave” (J-F9-G1-150623-F)	“I want to go from here, but I do not go because I have love and affection for the people of this area” (J-F5-G2-160623-S)	“I can’t say that now” (J-F9-G3-150623-F)
	High	“I don’t want to leave. May Allah take me to the grave from this place” (J-F*-G1-170623-F)	“We don’t want to go. My husband and I will stay here” (C-F2-G2-210523-S)	“I can live here all my life, I have no problem” (C-F2-G3-210523-S)

This table demonstrates participant quotes, exemplifying intellectual capital, and (Non-)migration decisions. The factors define the components of the analytical concept (see Fig. 1 and Table 1). Capabilities and aspirations are stratified on a scale from low to high (see Table 2 for all capabilities and aspirations across the five study sites)

^1^Interview ID (see Table S1 in the supplementary material)

Although the strict divide in traditional gender roles is becoming less prevalent in many households, women continue to be the primary caregivers of other household members, bearing responsibility for most homemaking tasks. As a result, during climate hazards such as floods or cyclones, the impact on homes directly translates into an increased burden on women. They are often tasked with preparing for and adapting to these events, ensuring the safety and well-being of their families. This responsibility extends beyond immediate actions; women also play a crucial role in passing on these adaptive strategies to their daughters. These daughters, in turn, are expected to assume the same burden when they marry, becoming responsible for their own families’ livelihoods during future climate hazards. This intergenerational transfer of knowledge not only underscores the critical role women play in household resilience but also highlights how deeply these responsibilities influence broader decisions, such as whether to remain in or migrate from hazard-prone areas, which vary across types of natural hazards.

## Discussion and outlook

### Summary of findings and relevance to the existing theories

This study explores women’s non-migration experiences across generations and sites in Bangladesh, highlighting the prevalence of voluntary and involuntary non-migration driven by a combination of personal, economic, societal, and climate-related factors. Male decisions largely dictate migration experiences despite women’s desire to move. However, many women expressed a low capability to move due to lacking financial capital and agency. Others remain attached to their current residence due to family responsibilities, place attachment, and fear of losing property. Place attachment is often driven by a deep emotional connection to the area and economic place-belongingness (Jennath & Paul, 2024), such as land ownership and agricultural employment that bind households to their residence. Moreover, in line with findings by Furlong et al. (2022), many women actively choose to stay to preserve family honor by adhering to societal norms. In many cases, this mindset becomes internalized, leading women to view immobility not as a constraint but as their customary way of life, with mobility not seen as a viable option.

Generational differences emerge, with third-generation participants aspiring to move for education or employment. At the same time, older generations often express aspirations to remain in their current homes due to health issues, attachment to their homes, or traditional gender roles. Participants’ deep attachment to their current residence stems from a sense of belonging, familiarity, and comfort associated with their home, family, and community, aligning with place attachment theories (Adams, 2016; Best et al. 2022). Traditional gender roles persist in many households, limiting girls’ education and mobility. While more third-generation girls are enrolled in school, societal expectations still hinder their pursuit of higher education. Our findings claim that some women aim to break the cycle of involuntary non-migration by sending their daughters away for better educational opportunities. In contrast, others continue the tradition of marrying off their daughters, perpetuating immobility through the next generation. Such findings reclaim the importance of gender and empowerment discourses related to climate change (Resurrección et al., 2019), echoing critical migration theorists’ assertions about how structural inequalities and barriers impede women’s migration aspirations (Furlong et al., 2022).

Findings also show that environmental hazards affect migration differently across regions, with cyclones in Khulna and Satkhira causing temporary or permanent displacement, while landslides in Chottogram rarely result in forced migration. Similarly, the frequent displacement caused by annual floods and river erosion in Jamalpur and Natore exacerbates socio-economic differences and insecurity, reflecting the heightened social vulnerability of marginalized households with limited resources and adaptive capacities. These patterns exemplify intersection of environmental change, power dynamics, and socio-economic structures. The differential impact of environmental hazards on migration across regions also reflects underlying political and economic factors, perpetuating vulnerabilities and amplifying challenges for women facing disproportionate climate impacts (Le, 2019).

Despite the risks, findings show that many households choose to return to their hometowns after extreme climatic events, influenced by factors such as place attachment, land ownership, and employment opportunities. The many households that choose to remain in their homes rely on intergenerationally passed-down adaptation strategies. Traditionally responsible for homemaking tasks, women play a crucial role in developing and transmitting their relevant intellectual capital, ensuring the resilience of their households in the face of ongoing risks. This intergenerational knowledge transfer is deeply intertwined with the gendered division of labor, reinforcing the non-migration decisions that allow families to maintain their livelihoods in these challenging environments. Cultivated intellectual knowledge on climate hazard resilience through intergenerationally transferred knowledge mediates the impact of environmental hazards on migration, as well as socio-economic status, access to resources, and female agency (Akhter et al., 2023; Furlong et al., 2022).

Overall, the findings underscore the need for a nuanced understanding of gender dynamics, intergenerational learning influences, and environmental vulnerabilities in shaping migration decisions and experiences, particularly for women, which is a novel empirical contribution to understanding women’s immobility despite climate risk.

### Reflection on the proposed analytical framework

Our conceptual framework outlines the societal and individual factors impacting individual-level migration outcomes within an intergenerational context. The framework employed in this study elucidates women’s (non-) migration decisions and strategies influenced by factors such as learning and knowledge transfer, individual attributes (including locality, intersectionality, and intergenerationality), environmental settings, and social, economic, political, and demographic factors, which collectively shape individual intellectual capital. This intellectual capital, in turn, affects an individual’s mobility capability and aspiration, resulting in states of non-migration. From the findings, it is evident that societal and environmental settings shape individual intellectual knowledge intersectionally and spatially across generations and genders. Further, environmental stress, including both slow- and rapid-onset events, can either incentivize migration by exacerbating livelihood challenges or hinder migration by impairing women's capability to move.

The framework pushes the boundaries of state-of-the-art environmental mobility frameworks by integrating a gendered lens and acknowledging the influence of intergenerational learning on migration outcomes. Intergenerational sociocultural traditions, knowledge, and expectations shape mobility decisions by perpetuating gender norms, roles, and ideologies. These norms contribute to unequal mobility capabilities and aspirations among individuals. The synthesis of these factors yields four categories of women’s mobility: voluntary and involuntary migration or non-migration. Overall, the framework provides a comprehensive understanding of the complex interplay of factors influencing migration outcomes within an intergenerational context, as exemplified empirically.

### Future research avenues

Based on the findings presented in the study and the novelty of employing intergenerational learning in an environmental mobility framework, there are several promising avenues for future research. Exploring how intersecting identities such as class, ethnicity, and age intersect with gender to shape women’s non-migration experiences could offer a nuanced understanding of how multiple forms of discrimination and privilege intersect with mobility decisions. Similarly, longitudinal studies tracking changes in women’s mobility aspirations and experiences over time could identify trends and patterns in non-migration behavior amid evolving socio-environmental conditions and policy interventions. This study may lead to comparative studies across different regions and countries to examine how varying sociocultural, economic, and environmental contexts shape women’s non-migration experiences. Such studies could provide insights into the generalizability of findings and the importance of context-specific factors. Besides this comparative aspect, additional underlying mechanisms and tools can be integrated into the employed framework (e.g., pro-environmental behavior, cognitive tools) to increase its holistic understanding and effectiveness in addressing environmental mobility decisions.

Additionally, policy analysis is vital to assess the effectiveness of existing policies and interventions in addressing the structural inequalities and barriers to women’s mobility, paving the way for gender-responsive policy interventions supporting women's agency and resilience against climate risks. This could include examining the effectiveness of interventions such as livelihood diversification, disaster risk reduction, and access to education and healthcare in enhancing women’s resilience and mobility options by helping co-design contextually relevant interventions and empowering communities to advocate for women’s rights and priorities. These efforts deepen understanding of cultural, social, and psychological factors driving women’s non-migration decisions despite climate risk, reinforcing the design of inclusive policies promoting gender equity, resilience, and sustainable development.

## Supplementary Information

Below is the link to the electronic supplementary material.

Supplementary file1 (DOCX 52 KB)

Supplementary file2 (DOCX 84 KB)

## Data Availability

The data supporting this study's findings are available upon reasonable request from the corresponding author.

## Footnotes

1 All names in this paper are fictitious, to protect the participant’s privacy and ensure anonymity. Participant’s real names are only known to the researchers.

## Code availability

Supplementary materials supported.
