# Supplementary material for: Intergenerational grounding of women’s environmental non-migration
Source: Popul Environ. 2025 Jan 22;47(1):7. doi: 10.1007/s11111-025-00475-w (PMC11928378; doi:10.1007/s11111-025-00475-w)
Supplement: Supplementary file 1 — Supplementary file1 (DOCX 52 KB) [file 11111_2025_475_MOESM1_ESM.docx]

Intergenerational Grounding of Women’s Environmental (Non-)migration

# Supplementary-1

**Table S1**

*Participant characteristics*

| **Interview ID** | **Study district** | **Age^a^** | **Religion** | **Main occupation** | **Economic conditions^e^** | **Mobility (migration or non-migration) motivations and challenges** |
| --- | --- | --- | --- | --- | --- | --- |
| C-F1-G1-0523-M | Chottogram | N/A | Islam | Homemakerᵇ | Financial hardship/socioeconomic disadvantage | Voluntary non-migration |
| C-F1-G2-0523-M | Chottogram | 32 | Islam | Homemaker | Financial hardship/socioeconomic disadvantage | Voluntary non-migration (“I think I am very good here”) |
| C-F2-G1-210523-S | Chottogram | N/A | Islam | Retiredᶜ | Financial hardship/socioeconomic disadvantage | Voluntary non-migration (family is here) |
| C-F2-G2-210523-S | Chottogram | 40 | Islam | Homemaker | Financial hardship/socioeconomic disadvantage | Voluntary non-migration (“I want to stay here… We don’t want to go”) |
| C-F2-G3-210523-S | Chottogram | 20 | Islam | Student | Financial hardship/socioeconomic disadvantage | Voluntary non-migration (“I can live here all my life, I have no problem”) |
| C-F3-G1-210523-F | Chottogram | 59 | Islam | Homemaker | Financial hardship/socioeconomic disadvantage | Voluntary non-migration (“No one wants to leave their land”) |
| C-F3-G3-210523-F | Chottogram | 18 | Islam | Student | Financial hardship/socioeconomic disadvantage | Voluntary migration aspirations (grew up in Dhaka and wants to move back to study) |
| I-F1-G1-110523-F | Natore | 57 | Islam | Homemaker | Financial hardship/socioeconomic disadvantage | Involuntary non-migration (?) (“I can’t go even if I want to”) / Voluntary non-migration (“I am going to the grave after a few more days”) |
| I-F1-G2-110523-F | Natore | 35 | Islam | Factory worker and homemaker | Financial hardship/socioeconomic disadvantage (earns 1,000 takas or €8 per month) | Involuntary non-migration (“I wanted to leave this place […], but my son’s father refused to leave […] his paternal house”) |
| I-F1-G3-110523-F | Natore | 18 | Islam | Student | Financial hardship/socioeconomic disadvantage | Voluntary migration aspirations (“I want to go away […] I don’t like it here”) |
| I-F2-G1-120523-F | Natore | 60 | Islam | Homemaker | Financial stability/a comfortable level of relative economic prosperity | Voluntary non-migration (“Why should I leave my area and go somewhere else?”) |
| I-F2-G2-120523-F | Natore | 35 | Islam | Homemaker | Financial hardship/socioeconomic disadvantage (has many loans) | (In)voluntary non-migration (“No, there is income here. But the problem is I owe [money] to people […] that I must repay”) |
| I-F2-G3-120523-F | Natore | 18 | Islam | Student | Financial hardship/socioeconomic disadvantage | Voluntary non-migration (“I want to stay here. Where else would I like to stay?”) |
| I-F3-G1-110523-S | Natore | 73 | Islam | Farmerᵈ and homemaker | Financial hardship/socioeconomic disadvantage | Voluntary non-migration (“I will not go to any other place. Here is the end”) |
| I-F3-G2-110523-S | Natore | 53 | Islam | Homemaker | Financial hardship/socioeconomic disadvantage | Voluntary non-migration (“I want to spend my days by staying here”), past and future forced migration (“We have to go if the place went into the river”) |
| I-F3-G3-110523-S | Natore | 20 | Islam | Homemaker | Financial hardship/socioeconomic disadvantage | Involuntary non-migration (“if he [husband] wants to keep me here, he will keep”) |
| I-F4-G1-120523-S | Natore | 71 | Islam | Homemaker | Financial hardship/socioeconomic disadvantage | Voluntary non-migration (“I don’t go anywhere”) |
| I-F4-G2-120523-S | Natore | 35 | Islam | Homemaker | Financial hardship/socioeconomic disadvantage | Voluntary non-migration (“Whoever likes a place, does he want to go from there?”) |
| I-F4-G3-120523-S | Natore | 18 | Islam | Student | Financial hardship/socioeconomic disadvantage | Voluntary non-migration (“I feel good here”) |
| I-F5-G1-010523-M | Natore | 71 | Islam | Day laborer (sewing, fishing) and homemaker | Financial stability/a comfortable level of relative economic prosperity | Involuntary non-migration (“We wish to go to the city, but there’s no way. We have to stay here”) |
| I-F5-G2-010523-M | Natore | 46 | Islam | Homemaker | Financial stability/a comfortable level of relative economic prosperity | Voluntary non-migration (“I want to die here”) |
| I-F5-G3-010523-M | Natore | 22 | Islam | Homemaker | Financial stability/a comfortable level of relative economic prosperity | Voluntary non-migration (“I’m living here for a long time, so I have feelings for it”) |
| I-F6-G1-020523-M | Natore | N/A | Islam | Homemaker | Financial hardship/socioeconomic disadvantage (eats by begging) | Voluntary non-migration (“I don’t want to go”) |
| I-F6-G2-020523-M | Natore | 35 | Islam | Jute mill worker | Financial hardship/socioeconomic disadvantage | Voluntary non-migration (“It’s better here than there [Dhaka]”) |
| I-F6-G3-020523-M | Natore | 19 | Islam | Homemaker | Financial hardship/socioeconomic disadvantage | Voluntary non-migration |
| J-F1-G1-0523-M | Jamalpur | N/A | Islam | Retired | Financial hardship/socioeconomic disadvantage | Voluntary non-migration (“Don’t go anymore, live and die here”) |
| J-F1-G2-0523-M | Jamalpur | N/A | Islam | Homemaker | Financial hardship/socioeconomic disadvantage | Involuntary non-migration (“We don’t have [money], so we can’t go”) |
| J-F1-G3-0523-M | Jamalpur | 21 | Islam | Student | Financial hardship/socioeconomic disadvantage | Voluntary non-migration |
| J-F2-G1-0523-M | Jamalpur | 56 | Islam | Farmer and homemaker | Financial hardship/socioeconomic disadvantage | Involuntary non-migration (“If Allah gives us the ability to buy two acres of land, then we would move to another place”) |
| J-F2-G2-0523-M | Jamalpur | 35 | Islam | Homemaker | Financial hardship/socioeconomic disadvantage | Mobility decisions depend on her husband, who has authority over her |
| J-F2-G3-0523-M | Jamalpur | 19 | Islam | Student | Financial hardship/socioeconomic disadvantage | Voluntary non-migration |
| J-F3-G1-0523-M | Jamalpur | N/A | Islam | Retired | Financial hardship/socioeconomic disadvantage | Voluntary non-migration |
| J-F3-G2-0523-M | Jamalpur | N/A | Islam | Homemaker | Financial hardship/socioeconomic disadvantage | Voluntary non-migration |
| J-F3-G3-0523-M | Jamalpur | 21 | Islam | Student | Financial hardship/socioeconomic disadvantage | Voluntary non-migration |
| J-F4-G1-160623-S | Jamalpur | 64 | Islam | Retired | Financial hardship/socioeconomic disadvantage | Never considered migrating due to family ties and money issues |
| J-F4-G2-160623-S | Jamalpur | 46 | Islam | Homemaker | Financial hardship/socioeconomic disadvantage | Voluntary non-migration |
| J-F5-G1-160623-S | Jamalpur | 74 | Islam | Retired | Financial hardship/socioeconomic disadvantage | Never considered migrating due to family ties and money issues |
| J-F5-G2-160623-S | Jamalpur | 33 | Islam | Homemaker | Financial hardship/socioeconomic disadvantage | Sometimes consider migrating (“but I do not go because I have love and affection to the people of this area [and] there is no money”) |
| J-F6-G1-170623-S | Jamalpur | 67 | Islam | Retired | Financial hardship/socioeconomic disadvantage | Voluntary non-migration |
| J-F6-G2-170623-S | Jamalpur | 36 | Islam | Homemaker | Financial hardship/socioeconomic disadvantage | Involuntary non-migration (“Allah didn’t give us the ability”) |
| J-F6-G3-170623-S | Jamalpur | 20 | Islam | Student | Financial hardship/socioeconomic disadvantage | Migration aspirations (“When I go to study, I will leave here”) |
| J-F7-G1-160623-F | Jamalpur | 72 | Islam | Retired | Financial hardship/socioeconomic disadvantage | Involuntary non-migration (“How could I move with so many babies […] I was hungry also”) |
| J-F7-G2-160623-F | Jamalpur | 35 | Islam | Farmer and homemaker | Financial hardship/socioeconomic disadvantage (she is the only income earner for the household) | Voluntary non-migration (“we have more affection for the country. […] If I die, I will die in the country”) |
| J-F7-G3-160623-F | Jamalpur | 19 | Islam | Student | Financial hardship/socioeconomic disadvantage | Voluntary non-migration |
| J-F8-G1-170623-F | Jamalpur | 56 | Islam | Homemaker | Financial hardship/socioeconomic disadvantage | Her husband doesn’t want to go somewhere else and they don’t have the ability (involuntary non-migration) (“If we had the ability, we would’ve moved there”) |
| J-F8-G2-170623-F | Jamalpur | 40 | Islam | Homemaker | Financial hardship/socioeconomic disadvantage | Involuntary non-migration (“Even if I want to go anywhere, we can’t go”) |
| J-F8-G3-170623-F | Jamalpur | 18 | Islam | Student | Financial hardship/socioeconomic disadvantage | She would want to go, but it depends on a possible future marriage and if her family will buy land elsewhere |
| J-F9-G1-150623-F | Jamalpur | 77 | Islam | Day laborer (working in other people’s homes) and homemaker | Financial hardship/socioeconomic disadvantage | (In)voluntary non-migration (“We do not have money […] Also, I can't get rid of the affection for homeland”) |
| J-F9-G3-150623-F | Jamalpur | 19 | Islam | Student | Financial hardship/socioeconomic disadvantage | She would move once she would marry |
| K-F1-G1-0523-M | Khulna | N/A | Islam | Shop owner, fisherwoman, and homemaker | Financial hardship/socioeconomic disadvantage | Voluntary non-migration |
| K-F1-G2-0523-M | Khulna | N/A | Islam | Catching and selling livestock and homemaker | Financial hardship/socioeconomic disadvantage | Involuntary non-migration (“If I go to another place I can have a good life […] what will I gain if I live here?”) |
| K-F1-G3-0523-M | Khulna | 18 | Islam | Student | Financial hardship/socioeconomic disadvantage | She will go where her family goes |
| K-F2-G1-050523-T | Khulna | 75 | Islam | Retired | Financial stability/a comfortable level of relative economic prosperity | Voluntary non-migration (“Where will I go? The destination is the grave”) |
| K-F2-G2-050523-T | Khulna | N/A | Islam | Works in a pharmacy | Financial hardship/socioeconomic disadvantage | Involuntary non-migration and migration aspirations > wants to go but she has a debt and would need to rent a house if they would move |
| K-F2-G3-050523-T | Khulna | 18 | Islam | Student | Financial hardship/socioeconomic disadvantage | Doesn’t know |
| K-F3-G1-050523-T | Khulna | N/A | Islam | Retired | Financial hardship/socioeconomic disadvantage | Is too old and sick (doesn’t get out of bed) |
| K-F3-G2-050523-T | Khulna | 37 | Islam | Fired from job (currently unemployed), homemaker | Financial hardship/socioeconomic disadvantage | (In)voluntary non-migration (“I would have gone if I had money”) |
| K-F3-G3-050523-T | Khulna | 17 | Islam | Employed in a crab project | Financial hardship/socioeconomic disadvantage (earns 7,000 takas or €60 per month) | Will migrate with her family (“If my father and mother do, I will go. I don’t like it here”) |
| S-F1-G1-050523-M | Satkhira | 60 | Islam | Homemaker | Financial hardship/socioeconomic disadvantage (depends on children for income) | Voluntary non-migration (“This is my in-laws’ land, so I don’t wish to leave it”) |
| S-F1-G2-050523-M | Satkhira | 31 | Islam | Homemaker | Financial hardship/socioeconomic disadvantage | Voluntary non-migration |
| S-F1-G3-050523-M | Satkhira | 18 | Islam | Student | Financial hardship/socioeconomic disadvantage | Migration aspirations (“I want to go with my parents. After my studies are over, we’ll leave”) |
| S-F2-G1-060523-M | Satkhira | 68 | Islam | Homemaker | Financial hardship/socioeconomic disadvantage | Mobility decisions are made by her husband (“My husband will not go from here. He will not leave his father’s land, so I can’t go either”) |
| S-F2-G2-060523-M | Satkhira | 32 | Islam | Homemaker (used to work outside of the home but not anymore due to illness) | Financial hardship/socioeconomic disadvantage | Voluntary non-migration (“I don’t want to go, but if Allah takes us, there is nothing to do”) |
| S-F2-G3-060523-M | Satkhira | 17 | Islam | Student | Financial hardship/socioeconomic disadvantage | Doesn’t want to go but “If Allah wants my marriage in Dhaka, I will have to stay in that place even if I don’t want it” |
| S-F3-G1-050523-M | Satkhira | N/A | Islam | Homemaker | Financial hardship/socioeconomic disadvantage | Voluntary non-migration |
| S-F3-G2-050523-M | Satkhira | 38 | Islam | Farmer and homemaker | Financial hardship/socioeconomic disadvantage | Mobility decisions are made by her husband (“He doesn’t want to go”) |
| S-F3-G3-050523-M | Satkhira | 19 | Islam | Student | Financial hardship/socioeconomic disadvantage | Migration aspirations (“I will go permanently”) |
| S-F4-G1-050523-M | Satkhira | 59 | Islam | Homemaker | Financial hardship/socioeconomic disadvantage | Voluntary non-migration (“Affection for the people of this area”) + mobility decisions are made by her husband (“The men of the house did not want to go. As a woman I can’t go alone, even if I want to”) |
| S-F4-G2-050523-M | Satkhira | 47 | Islam | Homemaker | Financial hardship/socioeconomic disadvantage | Voluntary non-migration (“My body is ready to go [pass away]”) |
| S-F4-G3-050523-M | Satkhira | 17 | Islam | Student | Financial hardship/socioeconomic disadvantage | Migration aspirations (“After the SSC exam, there is no good place to study here”) |

a. In Bangladesh, many people may not know their exact age due to factors such as limited birth registration, cultural norms placing less emphasis on age tracking, and challenges in record-keeping. For this reason, not all participants were able to state their age (“N/A”) and many participants guessed their approximate age. Therefore, not all stated ages may be accurate.

b. Homemaker is a broad category of women responsible for (unpaid labor) tasks in and around the home such as cooking, cleaning, caregiving duties, animal husbandry (cows, goats, chicken), and taking care of their land.

c. The category “Retired” is both women who are retired from their paid as well as unpaid labor due to old age and/or sickness.

d. Farmers are women who are (informally) employed to farm other people’s lands and receive payment for their labor, as opposed to women who are taking care of their land and don’t receive payment for this (categorized as “homemaker”).

e. We have classified participants into two economic condition categories: i) Financial hardship/socioeconomic disadvantage. This category includes individuals or households facing financial challenges, limited access to resources, and economic instability. And ii) Financial stability/a comfortable level of relative economic prosperity. This category includes individuals or households with a stable financial situation, sufficient income, and access to resources that allow them to comfortably meet their basic needs and enjoy a certain level of economic security. The moderate financial condition category is excluded as individuals in a moderate financial condition can have experiences that could overlap with either category.
