# Supplementary material for: Intergenerational grounding of women’s environmental non-migration
Source: Popul Environ. 2025 Jan 22;47(1):7. doi: 10.1007/s11111-025-00475-w (PMC11928378; doi:10.1007/s11111-025-00475-w)
Supplement: Supplementary file 2 — Supplementary file2 (DOCX 84 KB) [file 11111_2025_475_MOESM2_ESM.docx]

Intergenerational Grounding of Women’s Environmental (Non-)migration

# Supplemenatry-2

**Table S2:** *Codebook*

| **Theme/ Concept** | **Component/factor** | **Root code** | **Category** | **Sub-code** | **Description** |
| --- | --- | --- | --- | --- | --- |
| Childhood |  | Child marriage |  | Against will | Encompassing all memories and reflections on the participant's childhood, including both events and sentiments. |
|  |  |  |  | Divorced |  |
|  |  |  |  | Fear |  |
|  |  |  |  | Sad |  |
|  |  |  |  | Violence |  |
|  |  | Memories |  | Arbitrary |  |
|  |  |  |  | Elephant attack |  |
|  |  |  |  | Floods |  |
|  |  |  |  | Liberation war |  |
|  |  |  |  | No storms |  |
|  |  |  |  | Storms |  |
|  |  | Hardships |  | No running water |  |
|  |  |  |  | Orphan |  |
|  |  |  |  | Poverty |  |
|  |  |  |  | Suffering |  |
| Climate events | Environmental settings – place vulnerability  Individual intellectual capital – intergenerational knowledge and learning transfer: intergenerational, intersectional, spatial | Cyclonic storms | Adaptive response | Awareness | Both slow- and rapid-onset events and structural weather conditions (e.g., monsoon) as directly experienced by participants. Includes descriptions of their experiences, witnessed/experienced consequences, sentiments, adaptive responses, and frequency of the events. |
|  |  |  |  | Cooking |  |
|  |  |  |  | Education |  |
|  |  |  |  | Elevation  Bed  Belongings |  |
|  |  |  |  | Praying |  |
|  |  |  |  | Preparations  None |  |
|  |  |  |  | Protection from Sundarbans |  |
|  |  |  |  | Rebuild and repair |  |
|  |  |  |  | Using boats |  |
|  |  |  |  | Warning |  |
|  |  |  |  | Work in other places |  |
|  |  |  | Consequences | Absence of Basic amenities  Kerosine  Drinking water  Facilities  Food  School  Work and income |  |
|  |  |  |  | Aid  Chairman  Each other  Government  NGO  None |  |
|  |  |  |  | Catching fish |  |
|  |  |  |  | Damage  Human-made structures  Natural environment  No personal structure damage  Reparations  None |  |
|  |  |  |  | Deaths |  |
|  |  |  |  | Disease |  |
|  |  |  |  | Flooding (storm surge)  Dam breaking |  |
|  |  |  |  | Fuel issues |  |
|  |  |  |  | Harm |  |
|  |  |  |  | Hunger |  |
|  |  |  |  | Livestock |  |
|  |  |  |  | Mud |  |
|  |  |  |  | Quarrel |  |
|  |  |  |  | Water  Contamination  Height  Logging  Salination |  |
|  |  |  |  | Wind |  |
|  |  |  | Direct experience |  |  |
|  |  |  | Education |  |  |
|  |  |  | Frequency | Annual |  |
|  |  |  |  | Decrease |  |
|  |  |  |  | Duration |  |
|  |  |  |  | Increase |  |
|  |  |  | Mobility | Cyclone shelter  Issues |  |
|  |  |  |  | Forced |  |
|  |  |  |  | Immobility  Don’t go to shelter  No shelters  Trapped  Voluntary |  |
|  |  |  |  | Living on the streets |  |
|  |  |  |  | Permanent migration |  |
|  |  |  |  | Seeking safety |  |
|  |  |  |  | Stay |  |
|  |  |  | River dam |  |  |
|  |  |  | Sentiments | Fear |  |
|  |  |  |  | Happiness |  |
|  |  |  |  | No fear |  |
|  |  |  |  | Nothing to do about it |  |
|  |  |  |  | Sadness |  |
|  |  |  |  | Suffering |  |
|  |  | Drought | Adaptive response | Aid |  |
|  |  |  |  | Diversifying income |  |
|  |  |  |  | False ceiling |  |
|  |  |  |  | Fan |  |
|  |  |  |  | Fetching water |  |
|  |  |  |  | Installing water pumps |  |
|  |  |  |  | Irrigation |  |
|  |  |  |  | None |  |
|  |  |  |  | Open space |  |
|  |  |  |  | Pesticides |  |
|  |  |  | Consequences | Damage |  |
|  |  |  |  | Difficulties working |  |
|  |  |  |  | Disease |  |
|  |  |  |  | Famine  Burning vegetation  Plants die |  |
|  |  |  |  | Illness |  |
|  |  |  |  | Loss of income |  |
|  |  |  |  | Suffering |  |
|  |  |  |  | Water evaporation |  |
|  |  |  |  | Water scarcity |  |
|  |  |  | Coping strategies | Income streams |  |
|  |  |  |  | Praying |  |
|  |  |  |  | Temporality |  |
|  |  |  | Frequency | Annual |  |
|  |  |  |  | Duration |  |
|  |  |  |  | Increased frequency |  |
|  |  |  |  | Increased severity |  |
|  |  |  |  | No knowledge |  |
|  |  |  | Past |  |  |
|  |  |  | Staying in place |  |  |
|  |  | Earthquakes | Disruption |  |  |
|  |  |  | No harm |  |  |
|  |  |  | Timestamps |  |  |
|  |  |  | Water and gas shut off |  |  |
|  |  | Flood | Adaptive response | Aid  Chairman  Each other  Government  Journalists  Medical  NGO  None |  |
|  |  |  |  | Aspirations |  |
|  |  |  |  | Borrowing money |  |
|  |  |  |  | Building rafts |  |
|  |  |  |  | Elevation  Bathroom  Belongings  Cooking  Food  Furniture  Beds  Table  House  Livestock  Platform  Shops |  |
|  |  |  |  | Getting water |  |
|  |  |  |  | Knowledge gap |  |
|  |  |  |  | Mobility  Forced  Going to dry places  Lost job  On the streets  Permanent migration  Return  Still go to work |  |
|  |  |  |  | Preparations  Food for livestock  None |  |
|  |  |  |  | Using boats |  |
|  |  |  |  | Warnings |  |
|  |  | Flood | Consequences | Absence of  Aid  Boats  Crops  Drinking water  Facilities  Food  Difficult to cook  Eating less on purpose  Fish  More fish before  Only dry foods  Rations  Running water  Contamination  School  Teachings on preparations  Work |  |
|  |  |  |  | Catching fish |  |
|  |  |  |  | Clear roads |  |
|  |  |  |  | Damage  Not so much  Rebuilding  No money |  |
|  |  |  |  | Restorations |  |
|  |  |  |  | Death |  |
|  |  |  |  | Disease  Go to the hospital  No medicines |  |
|  |  |  |  | Emotions  Fear  Suffering  Unsafe in shelters |  |
|  |  |  |  | Falling in water |  |
|  |  |  |  | Harvest |  |
|  |  |  |  | Immobility  Helping with household chores  Likes staying at home  Too young to understand  No shelter  Nowhere to go to  Voluntary |  |
|  |  |  |  | Livestock |  |
|  |  |  |  | Losing belongings |  |
|  |  |  |  | Muddy |  |
|  |  |  |  | People most affected |  |
|  |  |  |  | Snakes |  |
|  |  |  |  | Stench |  |
|  |  |  |  | Theft |  |
|  |  |  |  | Water  Contamination  Decreased  Dragging down  Height  No high ground  Water inside |  |
|  |  | River bank erosion | Frequency | Annual |  |
|  |  |  |  | Decrease |  |
|  |  |  |  | Duration |  |
|  |  |  |  | Increase |  |
|  |  |  |  | Prediction |  |
|  |  |  |  | Unpredictable |  |
|  |  |  | Past | Past experiences |  |
|  |  |  |  | Teaching and learning  Knowledge gap |  |
|  |  |  | River dam |  |  |
|  |  |  | Sentiments | Stressful |  |
|  |  | Landslide | Adaptive response | Place attachment |  |
|  |  |  | Annual |  |  |
|  |  |  | Collapse |  |  |
|  |  |  | Consequences | Compensation |  |
|  |  |  |  | Damage |  |
|  |  |  |  | Death |  |
|  |  |  | No indication |  |  |
|  |  |  | Not at risk |  |  |
|  |  |  | Other areas |  |  |
|  |  |  | Prior problem |  |  |
|  |  | Monsoon | Adaptive response | Preparations |  |
|  |  |  | Bad roads |  |  |
|  |  |  | Consequences | Inundation |  |
|  |  |  |  | Trapped inside  No work |  |
|  |  |  | Flow tide |  |  |
|  |  |  | Water drains |  |  |
|  |  | River erosion | Adaptive response | Aid |  |
|  |  |  |  | Construction river dam |  |
|  |  |  |  | Migration |  |
|  |  |  |  | Moving belongings in advance |  |
|  |  |  |  | No relocation |  |
|  |  |  |  | Using convenient roads |  |
|  |  |  |  | Working during dry season |  |
|  |  |  | Consequences | Damage  Land  No exposure  Reparations  To crops |  |
|  |  |  |  | Death |  |
|  |  |  |  | Difficulties with cultivation |  |
|  |  |  |  | Forced migration |  |
|  |  |  |  | Immobility  No school |  |
|  |  |  |  | No work |  |
|  |  |  |  | Victims |  |
|  |  |  |  | Water  Height  Land becomes river  Logging |  |
|  |  |  | Frequency | Annual |  |
|  |  |  |  | Decrease  River dam |  |
|  |  |  |  | Duration |  |
|  |  |  |  | Increase |  |
|  |  |  | Human aggravation |  |  |
|  |  |  | Past occurrences |  |  |
|  |  |  | Soil sinking |  |  |
|  |  | Storm (unspecified) | Adaptive response | Building cement house |  |
|  |  |  |  | Praying |  |
|  |  |  |  | Preparations |  |
|  |  |  |  | TV forecast |  |
|  |  |  | Consequences | Damage  Reparations  Fruitless |  |
|  |  |  |  | Death |  |
|  |  |  |  | Debt |  |
|  |  |  |  | Flooding |  |
|  |  |  |  | Mobility |  |
|  |  |  |  | Staying inside |  |
|  |  |  | Coping mechanism | Religion |  |
|  |  |  | Frequency | Decrease |  |
|  |  |  | Sentiments | Happiness |  |
|  |  | Strong winds | Adaptive response | Cutting hills |  |
|  |  |  |  | Cutting trees  Turn off electricity |  |
|  |  |  | No adaptive response |  |  |
|  |  |  | Consequences | No electricity |  |
|  |  |  |  | No personal damage |  |
|  |  |  |  | Tree damage |  |
|  |  |  |  | Vulnerable houses  No help from chairman |  |
|  |  |  |  | Water level rise  Mode of trams |  |
| Economy and demographics | Collective engagement – drivers: social, economic, political, demographic | Employment sectors | Agriculture |  | Encompasses societal economic and demographic structures. These include economic sectors that participants (or according to their knowledge, broader society) are employed in. Also touches upon other demographic factors such as participant education levels and religion(s). |
|  |  |  | Industry |  |  |
|  |  |  | Services |  |  |
|  |  |  | Unpaid labor |  |  |
|  |  | Religion |  |  |  |
|  |  | Social cohesion |  |  |  |
| Family |  | Death |  |  | Encompasses information on the nuclear and extended family of the participants. Includes descriptions of their family members (education level, occupation, place of residence) and descriptions of dead family members. |
|  |  | Extended | Cousins |  |  |
|  |  |  | Grandchildren | Caretaking duties |  |
|  |  |  | Grandparents | Occupation |  |
|  |  |  | In-laws | Daughter-in-law gone |  |
|  |  |  |  | Dispute |  |
|  |  |  |  | Divorced |  |
|  |  |  |  | Parental role |  |
|  |  |  | Uncles | Mobility |  |
|  |  | Nuclear | Children | Bad relationship |  |
|  |  |  |  | Education |  |
|  |  |  |  | Housing |  |
|  |  |  |  | Marriage |  |
|  |  |  |  | Miscarriage |  |
|  |  |  |  | Occupation |  |
|  |  |  |  | Son missing |  |
|  |  |  |  | Unhappy |  |
|  |  |  | Parents | Absence |  |
|  |  |  |  | Bad relationship |  |
|  |  |  |  | Illness |  |
|  |  |  |  | Immobility |  |
|  |  |  |  | Occupation |  |
|  |  |  |  | Residence |  |
|  |  |  |  | Well off |  |
|  |  |  | Partner | Divorce |  |
|  |  |  |  | Ill |  |
|  |  |  |  | Marriage  Divorce |  |
|  |  |  |  | Occupation |  |
|  |  |  | Siblings | Education |  |
|  |  |  |  | Occupation |  |
|  |  |  |  | Place of residence |  |
|  |  |  | Stepchildren |  |  |
| Geographical (physical space) |  | Group conflict |  |  | Encompasses descriptions of human-made and natural changes to the landscape (participants' place of residence) over the years. Also includes participants' sentiments and weather patterns of their place of residence. |
|  |  | Changes | Anthropogenic | Access to goods |  |
|  |  |  |  | Agricultural technology |  |
|  |  |  |  | Biodiversity  Decrease  Increase |  |
|  |  |  |  | Cyclone center |  |
|  |  |  |  | Electricity  Solar |  |
|  |  |  |  | Hospital |  |
|  |  |  |  | Housing |  |
|  |  |  |  | Infrastructure |  |
|  |  |  |  | Irrigation system |  |
|  |  |  |  | Landscape deformation |  |
|  |  |  |  | Population growth |  |
|  |  |  |  | Power plant |  |
|  |  |  |  | Rail line |  |
|  |  |  |  | Running water |  |
|  |  |  |  | School |  |
|  |  |  |  | Tube wells  Submersibles |  |
|  |  |  |  | Agriculture |  |
|  |  |  | Satisfaction of area | Dissatisfaction |  |
|  |  |  |  | Facilities |  |
|  |  |  |  | Social cohesion |  |
|  |  |  | Weather conditions | Cold winters |  |
|  |  |  |  | Heat |  |
|  |  |  |  | No natural hazards |  |
|  |  |  |  | No perceived climate change |  |
| Individual (socio-)economic position | Collective engagement – drivers: economic, demographic | Economic position | Cultivation |  | Encompasses factors that influence participants' (socio-)economic position in society (education, occupation, physique). |
|  |  |  | Hardships |  |  |
|  |  |  | Land-property ownership | Built own house |  |
|  |  |  |  | Inheriting land |  |
|  |  |  |  | Leasing land |  |
|  |  |  |  | No house |  |
|  |  |  |  | No land |  |
|  |  |  |  | No property ownership |  |
|  |  |  |  | Property claims |  |
|  |  |  |  | Various houses |  |
|  |  |  | Livestock |  |  |
|  |  |  | Occupation | Income |  |
|  |  |  |  | Would work given the opportunity |  |
|  |  |  | Rich family |  |  |
|  |  |  | Well-of |  |  |
|  |  |  | Education |  |  |
|  |  | Physique | Play sports |  |  |
| Intergenerational knowledge transfer | Individual intellectual capital – intergenerational knowledge and learning transfer | No story transmission | Climate events | Cyclones | Includes descriptions of story transmissions from older family members to participant and from the participant to the younger generation(s). Also includes reflections on the absence of such stories. |
|  |  |  |  | Drought |  |
|  |  | Story transmission | Childhood memories |  |  |
|  |  |  | Climate events | Adaptive response |  |
|  |  |  |  | Cyclones |  |
|  |  |  |  | Drought |  |
|  |  |  |  | Flood |  |
|  |  |  |  | Hurricane experience |  |
|  |  |  |  | Monsoon |  |
|  |  |  |  | River erosion |  |
|  |  |  | General experiences | Fishing |  |
|  |  |  |  | Housing |  |
|  |  |  |  | Illness |  |
|  |  |  |  | Moving with in-laws |  |
|  |  |  |  | School |  |
|  |  |  |  | Struggle |  |
|  |  |  |  | To grandchildren |  |
| Mobility | Migration continuum – migration/non-migration decisions | Imaginaries | Aspirations | Depends | Encompasses (im)mobility decisions and actions. These include future mobility aspirations for the self and the participant's children. Further includes the incapability and/or unwillingness to move and past mobilities. |
|  |  |  |  | Desires |  |
|  |  |  |  | Move |  |
|  |  |  |  | No idea |  |
|  |  |  |  | Stay |  |
|  |  |  |  | To her children |  |
|  |  |  |  | Traveling |  |
|  |  |  |  | Twofold |  |
|  |  | Immobility (stay) | Incapability (forced) | Bound to family |  |
|  |  |  |  | Bound to husband |  |
|  |  |  |  | Health issues |  |
|  |  |  |  | Losing property |  |
|  |  |  |  | No connections |  |
|  |  |  |  | No financial resources |  |
|  |  |  |  | No place to go to |  |
|  |  |  |  | N sufficient education |  |
|  |  |  | Unwillingness (voluntary) | Acceptance |  |
|  |  |  |  | Burden to others |  |
|  |  |  |  | Dislike living in the city  Absence of nature  Air pollution  Crowded  Feels uncomfortable  Food quality  Misses family  Noise nuisance |  |
|  |  |  |  | Environmental stress everywhere |  |
|  |  |  |  | No benefit to moving |  |
|  |  |  |  | No consideration of migration |  |
|  |  |  |  | No emotional attachment |  |
|  |  |  |  | Place attachment  (Cheap) housing  Birthplace  Family  Food  Good facilities  Job  Nature  Peaceful  Pleasant temperature  Safety  Self-sufficiency  Social cohesion |  |
|  |  | Mobility (move) | Forced | House demolished |  |
|  |  |  |  | Living with in-laws  Grew up elsewhere  Same village |  |
|  |  |  |  | Moving back home  Difficulties adjusting  High living costs |  |
|  |  |  |  | Moving due to natural hazard |  |
|  |  |  |  | Moving with husband |  |
|  |  |  |  | Moving with parents |  |
|  |  |  |  | Sending children away |  |
|  |  |  | Voluntary | Abroad |  |
|  |  |  |  | City |  |
|  |  |  |  | For work |  |
|  |  |  |  | Living in daughter’s house |  |
|  |  |  |  | Moving between places  Husband |  |
|  |  |  |  | Others |  |
|  |  |  |  | Traveling |  |
|  |  |  |  | Visits family in city |  |
| Sentiments |  | Personal feelings | Negative | Injustice | Includes general sentiments, feelings, emotions, and life reflections of participants. |
|  |  |  |  | Sadness |  |
|  |  |  |  | Suffering |  |
|  |  |  |  | Survival |  |
|  |  |  | Neutral | Aspirations for children |  |
|  |  |  |  | Hereafter |  |
|  |  |  |  | Insensitive to natural events |  |
|  |  |  |  | No dwelling on past |  |
|  |  |  |  | Religious |  |
|  |  |  | Positive | Happiness |  |
|  |  |  |  | Peace |  |
| Social issues | Collective engagement – drivers: social, economic, political, demographic | Aid and development | Child marriage | Familial | Encompasses broader (structural) societal issues which (in)directly influence the participants' livelihood and their position in society. Also includes (foreign/external/national/local) aid and development strategies and implementations. |
|  |  |  | Corruption |  |  |
|  |  |  | Education system | Availability |  |
|  |  |  |  | Costs |  |
|  |  |  |  | Dropping out at a young age |  |
|  |  |  |  | Hunger |  |
|  |  |  |  | Literacy |  |
|  |  |  |  | No priority (other chores) |  |
|  |  |  |  | Quality |  |
|  |  |  |  | Regrets not studying |  |
|  |  |  |  | Stopping after marriage |  |
|  |  |  | Food (in)security | Cost |  |
|  |  |  |  | Nutrition |  |
|  |  |  |  | Ration card |  |
|  |  |  |  | Scarcity |  |
|  |  |  |  | Soil quality degradation |  |
|  |  |  |  | Water shortage |  |
|  |  |  | Gender inequality | Agency |  |
|  |  |  |  | Children |  |
|  |  |  |  | Education |  |
|  |  |  |  | Equality |  |
|  |  |  |  | Housing |  |
|  |  |  |  | Income |  |
|  |  |  |  | Marriage |  |
|  |  |  |  | Mobility |  |
|  |  |  |  | Work |  |
|  |  |  | Healthcare | Access |  |
|  |  |  |  | Animal attacks |  |
|  |  |  |  | Costs |  |
|  |  |  |  | Diseases |  |
|  |  |  |  | Heat |  |
|  |  |  | Housing | Congestion |  |
|  |  |  |  | Costs |  |
|  |  |  |  | Destruction |  |
|  |  |  |  | Electricity |  |
|  |  |  |  | Poor living conditions |  |
|  |  |  |  | Territorial conflicts |  |
|  |  |  | Infrastructure | Transportation |  |
|  |  |  | Poverty | Aid  Absence  Distribution  Financial compensation  Monetary support  Supported by children  Tangible support |  |
|  |  |  |  | Big household to support |  |
|  |  |  |  | Debt |  |
|  |  |  |  | Discrimination |  |
|  |  |  |  | Dowry |  |
|  |  |  |  | Financial crisis |  |
|  |  |  |  | High cost of living |  |
|  |  |  |  | Immobility |  |
|  |  |  |  | Income equality |  |
|  |  |  |  | Low or no income |  |
|  |  |  |  | No clothing |  |
|  |  |  |  | No employment opportunities |  |
|  |  |  |  | Religious coping |  |
|  |  |  | Security | Assault |  |
|  |  |  | Segregation |  |  |
|  |  |  | WASH | (Personal) sanitation and hygiene |  |
|  |  |  |  | Water  Access  Contamination and harm  Filtration  Leakage  No running water  Salination  Scarcity |  |
